# Rubella Immunity in Women of Childbearing Age, Eight Years After the Immunization Program in Iran

**DOI:** 10.5812/ircmj.10431

**Published:** 2014-07-05

**Authors:** Rahim Barari Sawadkohi, Amin Zarghami, Fatemeh Izadpana, Mohammad Pournasrollah

**Affiliations:** 1 Infectious Diseases Research Center, Amirkola Children Hospital, Babol University of Medical Sciences, Babol, IR Iran; 2 Student Research Committee, School of Medicine, Babol University of Medical Sciences, Babol, IR Iran; 3 Non-Communicable Pediatric Diseases Research Center, Amirkola Children Hospital, Babol University of Medical Sciences, Babol, IR Iran

**Keywords:** Rubella, Congenital Rubella Syndrome, Immunity, Vaccination

## Abstract

**Background:**

Rubella is a viral disease with a worldwide distribution. Mass vaccination campaigns have increased the vaccine coverage in the world with substantial impact on reduction of rubella infections. In Iran, the national measles-rubella campaign, targeting individuals 5-25 years old, was initiated in 2003 and mass childhood vaccination against measles, rubella and mumps has continued ever since.

**Objectives:**

The aim of this study was to evaluate the efficacy of routine vaccination on rubella immunity among women of childbearing age in Babol, north of Iran.

**Patients and Methods:**

This cross-sectional study was conducted on 812 women of childbearing age living in Babol, north of Iran, in 2011. Twelve samples were excluded from the study because of inadequate sera amounts. Serum samples were examined for presence of rubella-specific IgG antibodies by means of quantitative ELISA.

**Results:**

From a total of 800 samples in this study, rubella IgG seropositivity was seen in 786 (98.3% [95% CI = %97.5-%99.1]) cases. The maximum IgG seropositivity (99.2%) was seen in the age group of 21-25 years old and the lowest immunity (87.7%) was in the group of above 30 years old.

**Conclusions:**

Our data indicated that the rate of seropositivity to rubella virus in our population was high, suggesting that vaccination has been successful in Babol, reducing the likelihood of congenital rubella infection.

## 1. Background

Rubella, commonly known as German measles, is a mild acute viral disease with exanthematous manifestations such as rash and lymphadenopathy, which typically affects children. Its major clinical importance is associated with transmission from the affected mother to the fetus via placenta. Rubella infection in pregnancy can result in miscarriage, stillbirth, or a baby born with congenital rubella syndrome (CRS). The highest risk of CRS is in countries with high susceptibility to rubella among women of childbearing age (1-3).

Eradication of CRS has been one of the leading goals of the World Health Organization (WHO) since 2000 (4). According to WHO reports, annually, 12000 infants are born with CRS in the eastern Mediterranean regional office (EMRO) region, including Iran (5). Previous local surveys in Iran during 1970s to 1990s revealed a range of immunity from 3% to 18.8% against rubella among Iranian girls and women (6).

In the second half of 2003, a public immunization program was conducted against measles and rubella in Iran. Over 33 million people, 5-25 years old, were vaccinated in the program with measles and rubella (MR) vaccines (measles, Edmonston Zagreb strain; rubella, RA27/3 strain [Serum Institute of India Ltd]). Since then, the trivalent vaccine of measles, rubella and mumps (MMR) has been routinely administered in children (7).

## 2. Objectives

The aim of this study was to evaluate the efficacy of routine vaccination on rubella immunity among women of childbearing age in Babol, north of Iran.

## 3. Patients and Methods

This cross-sectional study was conducted on 812 women of childbearing age, referred to the premarital diagnostic central laboratory in Babol, northern Iran, in 2011. The study protocol had previously been proved at the Research Ethics Committee of Babol University of Medical Sciences. All the childbearing age females were eligible to enter the study. After explaining the goal of the study, the informed consents were taken. Next, the blood samples were taken and transferred to the laboratory and stored at 4ºC in a refrigerator. Of 812 collected sera samples, 12 were excluded from the study because of inadequate volume; so, samples of 800 women were entered to the study. The sera samples were collected and assayed for rubella IgG antibodies, using a rubella IgG ELISA kit (IBL, Immunobiological Laboratories, Germany). Testing was performed according to the manufacturer’s instructions. Sensitivity and specificity of the rubella antibody detection tests were similar to values of 95%. As recommended by the manufacturer, based on the recommendations of the Rubella Subcommittee of the US National Committee for Clinical Laboratory Standards (NCCLS), we regarded anti-rubella IgG levels lower than 5 IU/mL as negative, and those between 5 and 9.9 IU/mL as equivocal. All samples with antibody levels below 10 IU/mL were analyzed a second time for conﬁrmation. According to the international agreement, rubella-speciﬁc IgG levels ≥ 10 IU/mL were considered to reﬂect protective immunity (8). Statistical analysis of the results was carried out using SPSS software version 18 (Chicago, IL, USA), using Fisher's exact test. A P value less than 0.05 was considered significant.

## 4. Results

The mean age of the participants was 21 ± 5.5 with a mode of 21 years. Of 800 sera samples collected, a total of 786 subjects were seropositive and 14 women were seronegative against rubella. According to our findings, 98.3% [95% CI = %97.5-%99.1] of females were immune to rubella virus. The date of birth/age was not available for 74 women. According to the analysis carried out using Fisher’s exact test on 726 cases in three age groups, there was a significant difference between rubella immunity and increment of age (P value < 0.001) (Table 1). A higher rate of rubella seropositivity (99.6%) was observed in the lower than 20 years and 20-30 years age groups, compared with those in the > 30 years age group.

**Table 1.** Rubella Seroprevalence in Different Age Groups

Age Group, y	Positive, No (%)	Negative, No (%)	No. of Participants
**< 20**	329 (98.8)	4 (1.2)	333
**20-30**	341 (99.1)	3 (0.9)	344
**> 30**	43 (87.8)	6 (12.2)	49
**Total**	713 (98.3)	13 (1.7)	726

## 5. Discussion

The findings of our study indicated that immunity against rubella virus in our population was 98.3%. Furthermore, in another study in Iran, the reported rate among women of childbearing age in Shiraz was 98.9%, one year after the nationwide vaccination. However, their sample size was less in comparison with ours (7). In a similar study in Netherlands, immunity to rubella infection was estimated 95% after nine years of mass vaccination (9). Similar findings were reported in Sweden, with a seroprevalence of 95.8% among 41637 individuals after 22 years of an MMR immunization program (10). Akkoyunlu et al. in northeastern Turkey revealed 73% (11) and Calimeri et al. in Italy measured 85.8% (12) immunity in their studies. According to the cost-effectiveness analysis performed, systematic vaccination costs three to four times less than elective vaccination (13).

Different factors could affect the rate of rubella immunity around the world. Greenaway et al. investigated the susceptibility rate of rubella-exposed populations among the migrants from six regions of the world, who had recently migrated to Montreal, Canada. Susceptibility to rubella infection varied from 6% in southern African women to 24% in those from east Asia. Susceptibility was dependent on sex, age, and region of origin; immigrant women were more susceptible to rubella (odds ratio: 1.7 [CI 1.2 to 2.6]) (14). This was consistent with findings of Ramos et al. in Spain (15).

Our findings indicated that higher rate of rubella seropositivity was observed in lower ages. Results from Ontario, USA, confirmed that younger women had the highest susceptibility to rubella and were significantly more likely to develop immunity if previously susceptible (16).

Serologic immunity studies are necessary to evaluate immunization policies for rubella control (17). This study documented the seroprevalence of rubella-specific IgG antibodies among women of childbearing age, following an extensive program of vaccination in northern Iran. Several local studies have been conducted to determine immunity to rubella in people of different age groups, especially in women of childbearing age. However, most of the studies within the country were carried out before mass vaccination programs. The high rate of immunity to rubella found in this study, even eight years after the program, indicated the efficacy of the vaccine as well as benefits of a childhood immunization policy. Effective vaccination programs are critical for elimination of rubella and prevention of CRS. According to our findings, rubella immunity was widespread in women of childbearing age in our study population. We suggest that similar studies could be conducted to assess the efficacy of the vaccination program in other Iranian provinces with larger study populations and better sampling methods, because one of the limitations of our study was that because of our sampling strategy, we were not capable of fully generalizing the findings to the target population.
